# BRAzil magnesium (BRAMAG) trial: a double-masked randomized clinical trial of oral magnesium supplementation in pregnancy

**DOI:** 10.1186/s12884-020-02935-7

**Published:** 2020-04-21

**Authors:** Carla Adriane Leal de Araújo, Joel Geoffrey Ray, José Natal Figueiroa, João Guilherme Alves

**Affiliations:** 1 Department of Pediatrics, Instituto de Medicina Integral Prof. Fernando Figueira (IMIP), Recife, Pernambuco Brazil; 2 Faculdade Pernambucana de Saúde (FPS), Recife, Pernambuco Brazil; 3 Departments of Medicine and Obstetrics and Gynaecology, St. Michael’s Hospital, University of Toronto, 30 Bond St, Toronto, ON M5B 1W8 Canada; 4 Department of Biostatistics, Instituto de Medicina Integral Prof. Fernando Figueira (IMIP), Recife, Pernambuco Brazil

**Keywords:** Pregnancy, Preterm birth, Newborn, Magnesium, Randomized clinical trial, Preeclampsia

## Abstract

**Background:**

There is conflicting evidence about the role of oral magnesium supplementation in the prevention of preterm birth and related adverse outcomes. The objective of this study was to compare magnesium citrate with placebo in the prevention of adverse perinatal and maternal outcomes among women at higher risk.

**Methods:**

This multicenter, double-masked, placebo-controlled randomized superiority clinical trial compared oral magnesium citrate 300 mg to matched placebo, from 12 to 20 weeks’ gestation until delivery. This trial was completed in three centers in northeastern Brazil. Eligible women were those with a singleton pregnancy and ≥ 1 risk factor, such as prior preterm birth or preeclampsia, or current chronic hypertension or pre-pregnancy diabetes mellitus, age > 35 years or elevated body mass index. The primary perinatal composite outcome comprised preterm birth < 37 weeks’ gestation, stillbirth > 20 weeks, neonatal death or NICU admission < 28 days after birth, or small for gestational age birthweight < 3rd percentile. The co-primary maternal composite outcome comprised preeclampsia or eclampsia < 37 weeks, severe gestational hypertension < 37 weeks, placental abruption, or maternal stroke or death during pregnancy or ≤ 7 days after delivery.

**Results:**

Analyses comprised 407 women who received magnesium citrate and 422 who received placebo. The perinatal composite outcome occurred among 75 (18.4%) in the magnesium arm and 76 (18.0%) in the placebo group – an adjusted odds ratio (aOR) of 1.10 (95% CI 0.72–1.68). The maternal composite outcome occurred among 49 (12.0%) women in the magnesium arm and 41 women (9.7%) in the placebo group – an aOR of 1.29 (95% CI 0.83–2.00).

**Conclusions:**

Oral magnesium citrate supplementation did not appear to reduce adverse perinatal or maternal outcomes in high-risk singleton pregnancies.

**Trial registration:**

ClinicalTrials.gov NCT02032186, registered January 9, 2014.

## Background

The World Health Organization (WHO) estimates that each year 15 million children are born preterm before 37 weeks’ gestation [1]. Prematurity is a leading cause of death in the neonatal period, and the second most common cause in children under age five years [1–4]. Preterm has immediate and long-term implications for the child, including cerebral palsy, learning difficulties and visual and hearing impairment [5–8]. The ensuing social and economic costs are great [9–11].

Brazil is among the top-10 countries with the highest number of premature births [1]. For example, of the nearly 3 million births registered in the country in 2015, about 10.8% were preterm [12]. In that same year, prematurity was the main cause of death in children under the age of five years [13]. Furthermore, in 2016, a national study identified that the North and Northeast regions of Brazil had the highest rates of preterm birth, at 13.0 and 12.9%, respectively [14]. Factors associated with prematurity in Brazil are higher social vulnerability -- low income, adolescent pregnancy and limited high school education – as well as inadequate prenatal care, a high rate of cesarean delivery and preeclampsia [15].

Magnesium deficiency has been implicated in adverse maternal and perinatal outcomes. Moreover, magnesium deficiency has been associated with an increased risk for gestational and adverse perinatal outcomes, such as gestational hypertensive syndromes, leg cramps and preterm birth [16–21]. Currently, there is no consensus about the daily requirements of magnesium during pregnancy [21, 22], with recommendations vary between 220 mg to 500 mg per day [22]. A recent meta-analysis of 10 randomized clinical trials (RCT), comprising 9090 pregnancies, evaluated oral magnesium supplementation during pregnancy [23]. Therein, oral magnesium did not significantly reduce the risk of preeclampsia, perinatal mortality or small for gestational age birthweight (SGA). Magnesium was associated with higher newborn Apgar scores and less hypoxic-ischemic encephalopathy. However, as only two of the 10 RCTs were considered to be of higher quality, the authors concluded that there is insufficient evidence as to whether oral magnesium supplementation in pregnancy is beneficial to mother or fetus [23]. Additionally, the effect of magnesium supplementation in the prevention of prematurity has had conflicting results, perhaps partly due to different the different forms of magnesium assessed in those trials [24–32].

Magnesium citrate has a high solubility in water, favoring greater intestinal tract absorption and bioavailability compared to other preparations [33]. Accordingly, the current study compared magnesium citrate with placebo in the prevention of adverse perinatal and maternal outcomes among women at higher risk.

## Methods

### Study design and setting

This multicenter, double-masked, placebo-controlled randomized superiority clinical trial was completed between November 2014 and January 2017. The study was conducted in three centers in Northeastern Brazil: Instituto de Medicina Integral Prof. Fernando Figueira (IMIP), Hospital Dom Malan (HDM), and Hospital Petronila Campos. The rate of preterm birth in Northeast of Brazil is about 13.0% 11.

### Participant selection

Eligible women where those with a singleton pregnancy, aged 18 to 45 years, and currently at a gestational age of 12 to 20 weeks, the latter based on the last menstrual period among women with a regular menstrual cycle, or by first-trimester pregnancy dating ultrasound. Additionally, eligibility required that a woman had at least one risk factor for preterm birth or an adverse perinatal outcome, namely, that related to a prior pregnancy (i.e., preterm delivery < 37 weeks, stillbirth at > 20^1/7^ weeks, placental abruption, preeclampsia or eclampsia, a liveborn infant with small for gestational age birthweight <3rd percentile (SGA) or a liveborn infant with birthweight < 2500 g), or that in the current pregnancy (i.e., nulliparity, chronic hypertension, type 1 or type 2 diabetes mellitus, maternal age > 35 years, pre-pregnancy maternal body mass index > 30 kg/m^2^, or currently smoking cigarettes). Study exclusion criteria were known uncontrolled hyperthyroidism or active parathyroid disease, a chronic diarrheal disease, a serum creatinine concentration > 1.1 mg/dL, or a serum magnesium concentration > 2.6 mg/dL at study entry. Before starting magnesium or placebo, serum creatinine and magnesium levels were measured.

Study recruitment was done at the time of a pregnant woman’s first consultation at each of the site’s outpatient care clinics. Every eligible woman received details about the study’s design and goals, and those who provided signed informed consent were included.

### Randomization procedure

Randomization was performed using a table of random numbers, prepared by a researcher who did not participate in the data collection. These numbers were generated in a computer by Random Allocation Software 2.0 program.

### Intervention

Consenting participants allocated to the active arm received a daily magnesium citrate capsule (300 mg elemental magnesium citrate per capsule), and those in the control arm received a daily placebo capsule identical to that in the active arm. While the original protocol plan was to administer magnesium citrate 150 mg twice daily [21], it was changed to 300 mg daily, before initiation of the study, to maximize patient compliance. Women were advised to take the capsule after breakfast, daily, until the end of the current pregnancy. Adherence to treatment was defined as the ingestion of at least 80% of the prescribed dose. The magnesium citrate and placebo capsules were manufactured by IMIP’s Department of Pharmacology, and were identical in colour and shape. The 300 mg daily dose of magnesium was chosen to achieve the daily nutritional needs, while minimizing the side effects (e.g., gastrointestinal or sleepiness) or toxicity of excess magnesium. The study medication packages were supplied to each local pharmacy with sequential numbers. Code break envelopes were supplied to the lead pharmacist, but were not available for the investigation team. Each medication pack was individually prescribed for each participant.

Adherence, adverse events, and clinical intercurrences were monitored by the research team at each routine prenatal visit, and every 60 days. Adequate adherence was defined as completion of at least 70% of the prescribed pill count. Anthropometric data and routine laboratory tests were also performed at these encounters. Pregnant women who did not attend their scheduled clinic visits were contacted by telephone and visited within their residence. Post-randomization losses were duly recorded, with the related reason. Criteria for unplanned study exist were clinical signs or symptoms reported by the patient due to the intake of the capsules, or cancellation of prenatal care at their local hospital.

### Outcomes

The primary perinatal composite outcome comprised preterm birth < 37 weeks’ gestation, stillbirth > 20 weeks, neonatal death or NICU admission < 28 days after birth, or small for gestational age birthweight < 3rd percentile. The co-primary maternal composite outcome comprised preeclampsia or eclampsia < 37 weeks, severe gestational hypertension < 37 weeks, placental abruption, or maternal stroke or death during pregnancy or ≤ 7 days after delivery. Preeclampsia was defined as an elevated systolic blood pressure > 140 mmHg or diastolic blood pressure > 90 mmHg, with ≥2+ proteinuria on urinary dipstick, and/or the HELLP Syndrome, while eclampsia was defined by hypertension and a new-onset seizure. Severe gestational hypertension was identified by non-proteinuric hypertension, with a systolic blood pressure > 160 mmHg or a diastolic blood pressure > 105 mmHg, and ≤ 1+ proteinuria. Maternal stroke was defined according to the current WHO definition (a clinical syndrome caused by focal or generalized brain injury that lasts more than 24 h or leads to death and has no other cause than vascular).

### Sample size and data analysis

At a sample size of 1000 women assigned to placebo and 2000 women assigned to Mg^++^ citrate, with power of 80% and a 2-sided *P*-value of 0.05, the study was equipped to detect at least a 22% relative risk reduction in the primary perinatal composite outcome, assuming a primary perinatal composite outcome rate of 18% in the placebo group and 14% in the Mg^++^ group.

Analyses followed an intention-to-treat principle. Bivariate analysis compared baseline characteristics using a chi-square or unpaired t-test between groups. Study outcomes were presented as rates, and unadjusted relative risks with 95% confidence intervals (CI). As statistically significant imbalances were seen for two baseline variables – gestational age at trial entry, and cohabitation with a partner – a logistic regression model included these variables, to generate adjusted odds ratios (OR) and 95% CIs, as planned [20].

Statistical significance was set at a *P*-value < 0.05. Data analyses were performed using Epi-Info version 7.1.3.10 (CDC, Atlanta) and STATA/SE 12.0.

### Patient and public involvement

No patients were involved in setting the research question or the outcome measures, nor were they involved in developing plans for or implementation of the study. No patients were asked to advise on interpretation or writing up of results.

## Results

Due to a regional Zika virus outbreak, the intended recruitment of 2000 women in the magnesium arm and 1000 women in the placebo arm was not achieved.

**Fig. 1:**
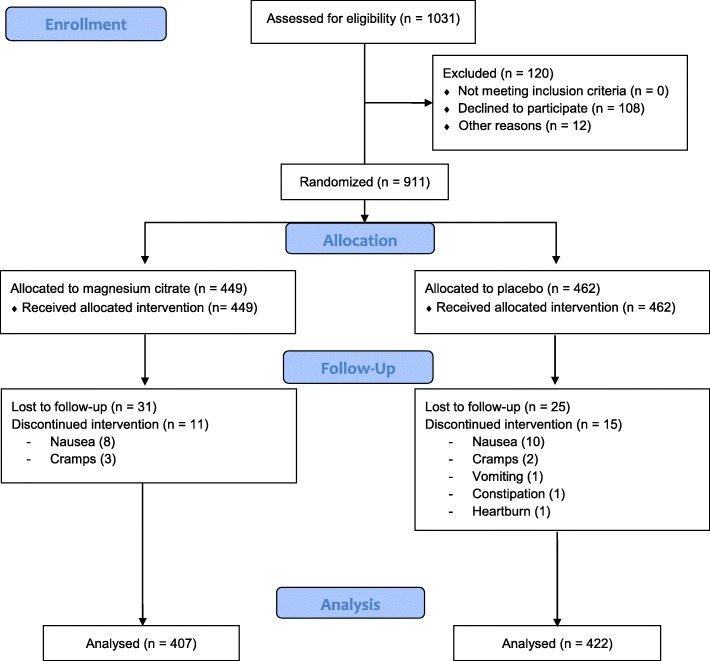
Flow diagram of participant recruitment, randomization and follow-up

Of the 1031 women who were eligible according to study entry criteria, 108 declined to participate and 12 were excluded for other reasons (Fig. 1). In total, 911 women were randomized: 449 to the magnesium citrate arm and 462 to the placebo arm. Of these, 31 (6.9%) and 25 (5.4%), respectively, did not complete the trial, because they voluntary abandoned prenatal care at IMIP (19 in the magnesium group and 21 in the placebo group), or moved to other services (12 in magnesium group and 11 in placebo group). Another 11 pregnant women in the intervention group and 15 in the placebo group had symptoms that led them to cease taking the study medication (Fig. 1).

Analyses comprised 407 women who received magnesium citrate and 422 who receivedplacebo. Intention-to-treat principle was used. The mean (SD) gestational age at study entry was 15.1 (3.6) and 15.6 (3.8) weeks, respectively. The two groups were similar according to their socio-demographic and clinical characteristics (Table 1). At trial entry, 194 women in the magnesium arm (47.9%), and 219 women in the placebo arm (52.1%) had a low serum magnesium concentration below 1.8 mg/dL.

**Table 1:** Characteristics of study participants at enrollment. All data are shown as a number (%) unless otherwise indicated

**Characteristic at enrollment**	**Magnesium citrate** **(*n*** **= 407)**	**Placebo** **(*n*** **= 422)**	***P*-value**
Mean (SD) maternal age, years	26.9 (5.4)	27.2 (5.8)	0.53
Mean (SD) gestational age at entry, weeks	15.1 (3.6)	15.6 (3.8)	0.05
Lives with partner	211 (51.8)	188 (44.5)	0.04
Completed schooling beyond grade 8	379 (93.3)	388 (91.9)	0.44
Currently employed	221 (54.3)	220 (52.1)	0.53
Alcohol use disorder	8 (2.0)	12 (2.8)	0.41
Current smoker	5 (1.2)	11 (2.6)	0.15
Current illicit drug use	3 (0.7)	5 (1.2)	0.73
Pre-pregnancy body mass index, kg/m^2^			
*< 18.5*	13 (3.2)	11 (2.6)	0.89
*18.5 to 24.9*	162 (39.8)	171 (40.7)	
*25.0 to 29.9*	136 (33.4)	146 (34.8)	
*≥ 30.0*	96 (23.6)	92 (21.9)	
Pre-pregnancy chronic hypertension	50 (22.1)	60 (24.4)	0.59
Gestational diabetes mellitus in a previous pregnancy	11 (4.9)	18 (7.3)	0.27
Preterm birth in a previous pregnancy	38 (9.6)	38 (9.3)	0.87
Mean (SD) serum magnesium concentration, mg/dL	1.8 (0.2)	1.8 (0.2)	0.94
Serum magnesium deficiency < 1.8 mg/dL	194 (47.9)	219 (52.1)	0.22
Mean (SD) serum creatinine concentration, mg/dL	0.5 (0.2)	0.5 (0.1)	0.80

The perinatal composite outcome occurred among 75 (18.4%) and 76 (18.0%) pregnancies, respectively (adjusted odds ratio [aOR] 1.10, 95% CI 0.72–1.68) (Table 2). The most common event within the perinatal composite outcome was preterm birth < 37 weeks’ gestation (9.3% vs. 9.0%, respectively) (adjusted OR 1.02, 95% CI 0.64 to 1.65).

**Table 2:** Primary perinatal and maternal outcomes comparing oral magnesium citrate vs. placebo

**Outcome**	**Magnesium citrate** **(*n* = 407)**	**Placebo** **(*n* = 422)**	**Unadjusted relative risk** **(95% confidence interval)**	**Adjusted odds ratio**^**a**^ **(95% confidence interval)**
**Primary perinatal composite outcome**	75 (18.4)	76 (18.0)	1.02 (0.76 to 1.36)	1.10 (0.72 to 1.68)
*Preterm birth < 37 weeks’ gestation*	38 (9.3)	38 (9.0)	1.04 (0.68 to 1.59)	1.02 (0.64 to 1.65)
*Stillbirth > 20 weeks’ gestation*	0 (0.0)	1 (0.2)	Not calculable	Not calculable
*Neonatal death < 28 days after birth*	4 (0.9)	10 (2.3)	0.41 (0.13 to 1.31)	0.39 (0.12 to 1.27)
*NICU admission < 28 days after birth*	12 (2.9)	9 (2.1)	1.38 (0.58 to 3.24)	1.38 (0.59 to 3.21)
*Small for gestational age birthweight < 3rd percentile*	21 (5.1)	18 (4.2)	1.21 (0.65 to 2.24)	1.37 (0.71 to 2.66)
**Primary maternal composite outcome**	49 (12.0)	41 (9.7)	1.24 (0.84 to 1.83)	1.29 (0.83 to 2.00)
*Preeclampsia < 37 weeks’ gestation*	24 (5.9)	20 (4.7)	1.24 (0.70 to 2.22)	1.25 (0.68 to 2.31)
Severe gestational hypertension < 37 weeks’ gestation	20 (4.9)	19 (4.5)	1.09 (0.59 to 2.01)	1.18 (0.62 to 2.26)
*Placental abruption*	9 (2.2)	21 (5.0)	0.44 (0.21 to 0.96)	0.43 (0.20 to 0.95)
*Maternal stroke during pregnancy or ≤ 7 days after birth*	0 (0.0)	0 (0.0)	Not calculable	Not calculable
*Maternal death during pregnancy or ≤ 7 days after birth*	1 (0.2)	0 (0.0)	Not calculable	Not calculable

^a^Odds ratios were adjusted for gestational age at trial entry and cohabitation with a partner, using logistic regression analysis

The maternal composite outcome occurred among 49 (12.0%) and 41 (9.7%) pregnancies, respectively (aOR 1.29, 95% CI 0.83–2.00) (Table 2). The most common events within the maternal composite outcome were preeclampsia < 37 weeks’ gestation (5.9% vs. 4.7%) and severe gestational hypertension < 37 weeks’ gestation (4.9% vs. 4.5%) (Table 2). Of note, the risk of placental abruption was lower in the magnesium group (9 events [2.2%]) compared to the placebo arm (21 events [5.0%]), equivalent to an adjusted OR of 0.43 (95% CI 0.20 to 0.95).

## Discussion

Apparently, in this study, oral magnesium citrate supplementation was not superior to placebo in the prevention of an array of adverse perinatal or maternal outcomes.

The 300 mg daily dose of magnesium citrate used herein approximated that recommended in pregnancy [22, 34]. A notable proportion of pregnant women (around 50%) had low serum levels of magnesium. Studies in India, Nigeria and Iran identified hypomagnesemia in pregnancy, at respective rates of 43.6, 16.2 and 13% [20, 35, 36]. Among 52 healthy pregnant women living in São Paulo, Brazil, despite having serum and red cell magnesium concentrations within normal limits, dietary intake was inadequate, and 39% had low urinary magnesium excretion [37]. An association between low maternal serum magnesium levels and preterm labour has been described previously [38].

The current findings are in keeping with some previously completed randomized clinical trials of oral magnesium supplementation for the prevention of preterm birth. Martin et al. studied 54 women at high risk of preterm birth, and observed that 1 g of magnesium gluconate, administered four times daily, was not effective for preventing preterm birth [27]. A double-masked RCT by Sibai et al. evaluated 374 high- risk pregnant women, and also did not show a significant difference in the risk of preterm birth with oral magnesium supplementation [25]. Arikan et al. studied 530 low risk pregnant women, and did not observe any reduction in the risk of preterm labour with oral magnesium citrate, 365 mg daily, started before 18 weeks’ gestation [39]. In a quasi-randomized trial of 568 pregnant women receiving magnesium aspartate or placebo, there was a significant reduction in preterm labor in the magnesium arm [24]. Another recent study of 180 pregnant women also found a significant reduction in preterm labor with magnesium in women with hypomagnesemia, using women with a normal serum magnesium concentration as controls [40].

The rate of preterm birth in Northeast Brazil is 12.9%, with a national rate of 11.1% [41]. Herein, the 9.2% rate of preterm birth was a little lower than expected, perhaps due to enhanced prenatal care offered within the context of a clinical trial, and the selection of more engaged study participants. There was only one stillbirth observed herein, and 10 neonatal deaths among controls vs. 4 deaths in the magnesium group. While a non-significant finding, future studies might consider the potential importance of this outcome related to magnesium supplementation. One study evaluated the risk of newborn hypoxic ischemic encephalopathy among 4082 pregnant mothers assigned oral magnesium stearate or placebo [30]. Although the rate of medication adherence was about 25%, no reduction in hypoxic ischemic encephalopathy was seen therein. Perinatal mortality was also higher in the treatment group (17 deaths) compared to the placebo arm (7 cases), but most deaths were attributed to congenital anomalies [30]. A recent meta-analysis of magnesium supplementation in pregnant women included 10 trials, with two judged to be of high quality [23]. Analysis restricted to these two trials showed no significant difference between the oral magnesium and control groups for perinatal mortality, SGA or preeclampsia [23].

A major study limitation herein was the lack of achievement of the target sample size. One major reason was an outbreak of microcephaly associated with Zika virus infection [42], which posed a major distraction to participant recruitment and the availability of resources. For example, there was concern among women about the use of medications in pregnancy, given that the etiology of the microcephaly outbreak was unclear at that time. In a post hoc calculation, using the currently achieved study sample size, to detect the expected 22% relative risk reduction in the primary perinatal composite outcome (i.e., an event rate of 14% in the magnesium group and 18% in the placebo group), our study had only 34.9% statistical power. Even so, the current data do provide important information about the efficacy of magnesium citrate supplementation in pregnancy, and the need to conduct further research in this area.

## Conclusions

Oral magnesium supplementation in pregnancy among higher-risk women does not seen to reduce preterm birth, or adverse perinatal or maternal outcomes.

## Data Availability

The data are available by establishing contact with the corresponding author at: (email: carla.leal@imip.org.br). The participants gave their consent for data sharing.

## Abbreviations

WHO: World Health Organization
SGA: small for gestational age birthweight
IMIP: Instituto de Medicina Integral Prof. Fernando Figueira
HDM: Hospital Dom Malan
RCT: Randomized clinical trial
CI: Confidence interval
OR: Odds ratio
SD: Standard deviation
CAM: Centro de Atenção à Mulher
